# Knowledge and practice of mothers towards sunshine exposure of their children in Ethiopia: a systematic review and meta-analysis

**DOI:** 10.1186/s12887-022-03281-7

**Published:** 2022-04-18

**Authors:** Eyasu Alem Lake, Birhanu Wondimeneh Demissie, Natneal Atnafu Gebeyehu, Gedion Asnake Azeze, Kelemu Abebe Gelaw, Robera Olana Fite, Lielt Gebreselassie Gebrekirstos, Tesfaye Yitna Chichiabellu, Mistire Teshome Guta

**Affiliations:** 1 School of Nursing, College of Health Science and Medicine, Wolaita Sodo University, Wolaita Sodo, Ethiopia; 2 School of Midwifery, College of Health Science and Medicine, Wolaita Sodo University, Wolaita Sodo, Ethiopia

**Keywords:** Knowledge, Practice, Sunshine, Sunlight, Mothers, Children, Ethiopia

## Abstract

**Background:**

Early morning sunlight exposure for infants is a good practice to prevent rickets and alleviate the problem of vitamin D deficiency. Rickets is a major public health problem in many countries especially in developing country including Ethiopia. As mothers are frontline person for their children who should know and practice about sunlight exposure, this systematic review and meta-analysis aimed to assess the pooled level of knowledge and practice towards sunlight exposure of their children among mothers in Ethiopia.

**Methods:**

PubMed, Google Scholar, Excerpta Medica database (EMBASE), Cochrane Library, Web of Science, and African Journal of Online (AJOL) were searched. The data were extracted using Microsoft Excel and analyzed using STATA version 14. Publication bias was checked by funnel plot and more objectively through Egger’s regression test, with *P* < 0.05 considered to indicate potential publication bias. The heterogeneity of studies was checked using I^2^ statistics. Pooled analysis was conducted using a weighted inverse variance random-effects model. Subgroup analysis was done related to geographic region and time. A leave-one-out sensitivity analysis was also employed.

**Result:**

A total of 8 studies with 2974 study participants for knowledge, nine studies with 3475 study participants for practice were used to estimate the pooled level of good knowledge and good practice of sunshine exposure among Ethiopian mothers. The overall estimated good level of knowledge and good practice towards sunshine exposure of their children among mothers was found to be 56.08% ((95% CI: 46.26 - 65.89%; I2 = 96.8%) and 55.632% (95%CI: 44.091 - 67.174%; I2 = 98.2%). Regional subgroup analysis showed that the pooled level of good practice in Amhara and Sidama regions found to be 54.41 and 58.32% respectively.

**Conclusion:**

Study findings showed mothers knowledge and practice towards sunshine exposure of children was quite low in Ethiopia. This study therefore recommends that interventions are needed to increase knowledge and practice of sunlight exposure. This study provides much needed significant evidence for making health-policy recommendations for this vulnerable population group.

## Introduction

The healing power of the sun and its use in medical treatment (heliotherapy) has extended back into ancient times starting from about1400 BC [1, 2]. In 1919, the first scientifically established health benefit of sun exposure was realized that sunlight used to prevent and cure rickets [3]. Recently being exposed to ultraviolet radiation has been a subject of epidemiological interest due to both its beneficial and detrimental effects. On the other hand, around 80–90% of vitamin D in human being is sunlight-derived production in the skin [3, 4]. Exposure of the body in bathing suit to one minimal erythemal dose (MED) (i.e. slight redness of the skin is equivalent to taking 10,000 to 25,000 IU vitamin D orally [4, 5].

Even if, Ethiopia is known as a country with 13 months of sunshine (12 months of 30 days each and 13th month of 5 days, which will be 6 days every leap year), children may be at increased risk for vitamin D deficiency because of the limited sun exposure due lack of adequate knowledge about the importance, timing and duration of sun exposure [6, 7].

Childhood rickets is common in low-and middle-income countries, especially the Indian subcontinent, Africa, and the Middle East where about 30% of children aged 0–5 years had at least one sign of nutritional rickets on clinical examination [8, 9] .

Rickets is quite prevalent (41%) among children in Ethiopia and is commonly associated with protein energy malnutrition, infectious diseases of various cause including respiratory tract, anemia, congestive heart failure and gastrointestinal tract risk recurrent diarrhea and mal absorption. The major cause of nutritional rickets in Ethiopian children is lack of exposure to sunshine and/or inadequate intake of vitamin D as its incidence is particularly high in slum children who live in crowded houses almost devoid of sunlight ([10–12], and). Low vitamin D status causes demineralization of skeleton and also associated with cardiovascular disease, certain cancers, cognitive decline, depression, diabetes mellitus, pregnancy complications and autoimmune diseases [13]. It contributes to infant mortality and morbidity and carries long-term consequences. Factors influencing caregiver behavior of exposing infants to sunshine, a simple preventive strategy, are not fully understood [14].

Health education to change maternal behavior to expose infants to sunshine was adopted as the main strategy to combat rickets in Ethiopia [10]. However, the implementation of the strategy has remained inconsistent. Besides, health education to change maternal behavior to expose infants to sunshine was adopted as the main strategy to combat rickets in early 1960s, yet the implementation of this strategy was inconsistent. Despite of all this efforts, lack sun light exposure continues to be the major risk factor of vitamin-D deficiency rickets in Ethiopia children ([10, 14, 15], and).

To our knowledge, this study is the first comprehensive national pooled estimate on Knowledge and Practice (KP) with respect to sunshine exposure among mothers in Ethiopia, and these have shown significant differences in KP between the various regions of the country. We therefore conducted this systematic review and meta-analysis to provide an estimate of KP with respect sunshine exposure of children among Ethiopian mothers for the purpose of improving program planning and interventions focused on health education targeting the importance of sun exposure during childhood period.

## Methods

### Searching strategy and source of information

This study was conducted to estimate the pooled level of KP of mothers towards sunshine exposure of their children in Ethiopia. We checked the DARE database (http://www.library.UCSF.edu) and the Cochrane library to ensure this had not been done before and to avoid duplication. We also checked whether there was any similar ongoing systematic review and meta-analysis in the PROSPERO database ((PROSPERO 2017:CRD42017074407); Available from http://www.Crd.york.ac.uk/PROSPERO_REBRANDING/displayrecord.asp?ID = CRD42017074407. These checks reassured us that there had been no previous similar studies undertaken.

All relevant and published researches in the following databases; PubMed, Google Scholar, Excerpta Medica database (EMBASE), Cochrane Library, Web of Science, and African Journal of Online (AJOL) were searched. We reviewed grey literature using Google. Unpublished studies were sought from the official website of an international and/or local organization or university.

The following core search terms or phrases were used; knowledge, awareness, practice, sunshine, rickets, vitamin D deficiency, children and mother. Search terms were pre-defined to allow a complete search strategy that included all-important studies. All fields within records and MeSH (Medical Subject Headings) and Boolean operators were used to search in the advanced PubMed search engine.

Notably, to fit with the advanced PubMed database the following search strategy was developed using different Boolean operators; ((((((((Knowledge[tw] OR Awareness[tw] OR Practice[tw])) OR (“Health Knowledge, Attitudes, Practice”[Mesh] OR “Practice Management, Veterinary”[Mesh] OR “Patient Medication Knowledge”[Mesh] OR “Knowledge Discovery”[Mesh] OR “Intraoperative Awareness”[Mesh] OR “Knowledge Management”[Mesh] OR “General Practice”[Mesh] OR “Practice Patterns, Nurses’“[Mesh] OR “Knowledge Bases”[Mesh] OR “Advanced Practice Nursing”[Mesh]))) AND (((Sunshine [tw] OR Sun light [tw])) OR “Sunlight”[Mesh])) AND ((Exposure [tw]) OR (“Pre-Exposure Prophylaxis”[Mesh] OR “Virtual Reality Exposure Therapy”[Mesh] OR “Post-Exposure Prophylaxis”[Mesh] OR “Inhalation Exposure”[Mesh] OR “Paternal Exposure”[Mesh] OR “Maternal Exposure”[Mesh] OR “Occupational Exposure”[Mesh] OR “Prenatal Exposure Delayed Effects”[Mesh] OR “Environmental Exposure”[Mesh] OR “Atmosphere Exposure Chambers”[Mesh] OR “Dental Pulp Exposure”[Mesh] OR “Exposure to Violence”[Mesh] OR “Dietary Exposure”[Mesh] OR “Radiation Exposure”[Mesh] OR “War Exposure”[Mesh] OR “Implosive Therapy”[Mesh] OR “Disease Notification”[Mesh]))) AND (((Newborn [tw] OR Infant [tw] OR children [tw])) OR (“Infant, Newborn, Diseases”[Mesh] OR “Infant, Newborn”[Mesh] OR “Infant Welfare”[Mesh] OR “Infant Health”[Mesh] OR “Respiratory Distress Syndrome, Newborn”[Mesh] OR “Infant Death”[Mesh] OR “Infant, Extremely Premature”[Mesh] OR “Transient Tachypnea of the Newborn”[Mesh] OR “Infant, Extremely Low Birth Weight”[Mesh] OR “Infant Formula”[Mesh] OR “Diapers, Infant”[Mesh] OR “Adult Children”[Mesh] OR “Disabled Children”[Mesh] OR “Dental Care for Children”[Mesh] OR “Infant, Very Low Birth Weight”[Mesh] OR “Infant Behavior”[Mesh] OR “Infant Equipment”[Mesh]))) AND (((Mother[tw] OR Women [tw])) OR (“Pregnant Women”[Mesh] OR “Kangaroo-Mother Care Method”[Mesh] OR “Women, Working”[Mesh] OR “Women”[Mesh] OR “Physicians, Women”[Mesh] OR “Battered Women”[Mesh] OR “Mother-Child Relations”[Mesh] OR “Dentists, Women”[Mesh] OR “Maternal-Fetal Relations”[Mesh] OR “Infectious Disease Transmission, Vertical”[Mesh] OR “Women’s Health Services”[Mesh] OR “Mothers”[Mesh] OR “Postpartum Period”[Mesh] OR “Achard-Thiers syndrome” [Supplementary Concept]))) AND ((Ethiopia [tw]) OR (“Ethiopia”[Mesh] OR “hemoglobin Ethiopia” [Supplementary Concept])). We reviewed studies that assessed KP on sunshine exposure through face to face interviews, self-administered questionnaires or checklist among mothers.

### Measurements of KAP

Knowledge: knowledge was assessed based on 10 questions about sunshine exposure that included the benefit, timing, duration and its drawback. Knowledge was defined as good if the respondents scored above the mean level.

Practice: practice was assessed by using 7 questions about the timing, frequency, duration and the condition of sunshine exposure and the respondent was categorized as showing good practice if she scored above the mean level.

### Reporting

The results of this review were reported in line with the Preferred Reporting Items for Systematic Review and Meta-Analysis statement (PRISMA) guideline [16].

### Eligibility criteria

All observational studies, including cross-sectional, case-control and cohort studies on KP of mothers towards sunshine exposure of their children were considered for this study. Those studies about KP of sunshine exposure among mothers which were published in English were included and there was no restriction on study period. The level of good knowledge and good practice was calculated using the data presented in the studies. Papers were excluded if they were: review articles, studies reporting confused data or with probable errors, studies without any information on the country and studies which were not able to fully access. An attempt was made to contact the corresponding authors using the email address or phone number as provided in the published articles.

### Study selection and extraction

Retrieved articles were exported to the reference manager software, Mendeley Desktop, and this was used to remove duplicate studies. Two independent reviewers screened the title and abstract. Any disagreement was handled based on established article selection criteria. Data were extracted using a standardized data extraction format prepared in Microsoft Excel by two independent reviewers. Any discrepancy during extraction was solved through discussion. The name of the first author, study area and region, the study year, study design, year of publication, study population, sample size, response rate and level of good knowledge and good practice were collected.

### Quality assessment

Three independent authors appraised the quality of the studies. The Joanna Briggs Institute (JBI) quality appraisal checklist was used [17]. When there was disagreement, all authors discussed and resolved the issue. The critical appraisal checklist had 8 parameters with options of “yes, no, unclear and not applicable.” The quality parameters included the following questions: (1) Were the criteria for inclusion in the sample clearly defined?, (2) Were the study subjects and the setting described in detail?, (3) Was the exposure measured in a valid and reliable way?, (4) Were objective, standard criteria used for measurement of the condition?, (5) Were confounding factors identified?, (6) Were strategies to deal with confounding factors stated?, (7) Were the outcomes measured in a valid and reliable way?, and (8) Was an appropriate statistical analysis used?. Studies were considered low risk if there was a score of 50% and above of the quality assessment indicators.

### Statistical analysis

The data were extracted using Microsoft Excel and analyzed by using STATA version 14 statistical software (stataCorp LP, 4905 Lakeway Drive, College Station, TX 77845, USA). Publication bias was checked by funnel plot and more objectively through Begg and Egger’s regression tests, with *P* < 0.05 considered to indicate potential publication bias [18, 19]. The presence of significant between-study heterogeneity was assessed using the Cochrane Q statistic. I^2^ was used to quantify between-study heterogeneity, in which a value of 0, 25, 50, and 75% indicated no, low, medium, and increased heterogeneity, respectively [20]. A forest plot was used to visualize the presence of heterogeneity. Since we found a high level of heterogeneity, we used a random-effect model for analysis to estimate Der Simonian and Laird’s pooled effect. Subgroup analysis was done by stud region. A leave-one-out sensitivity analysis was employed to see the effect of a single study on the overall meta-analysis estimate. The results were presented in the form of text, tables and figures.

## Result

### Search outcomes

**Fig. 1:**
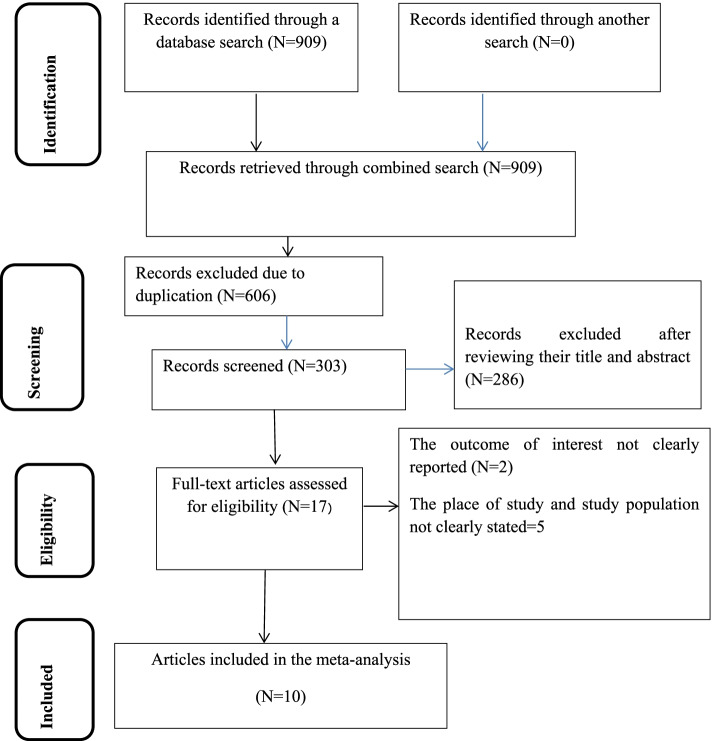
Schematic presentation of study selection for systematic review and meta-analysis of KP towards sunshine exposure of their children among mothers in Ethiopia

There were 909 articles retrieved using the electronic search. Of these articles, 606 were excluded due to duplication and 17 articles were fully accessed and assessed for qualification. Eventually, 10 articles met the eligibility criteria and were included in the final meta-analysis (Fig. 1).

### Characteristics of included studies

Eight articles which assessed knowledge, 9 articles which assessed practice. All of those which fulfilled the inclusion criteria were included in this systematic review and meta-analysis. All studies had a low risk during the quality assessment. Five studies were conducted in the Amhara region [10, 21–24], two in Sidama region [25, 26], one in Oromia [27] and one in Addis Ababa [28]. The other article on KP of mothers on sunshine exposure was nationwide study [29]. The earliest study was conducted by Tesfaye Getaneh in 1995 at Jimma town, Oromia region [27]. The recent study was from Addis Ababa which was conducted in 2020 [28]. Regarding the study design, all studies employed a prospective cross-sectional study design. The sample size ranges from 105 to 660, and the response rate ranged from 84.3 to 100%. The highest level of good knowledge (76%) was recorded in a study from Oromia region and the lowest (40%) was recorded in a study conducted in Amhara region. There was a high level of good practice recorded in a study done in Oromia region which was 80.1% (Table 1).

**Table 1:** Characteristics of studies included in the systematic review and meta-analysis on level of knowledge and practice of mothers towards sunshine exposure of their children in Ethiopia

*Authors name*	Study year	Date of accessed	Study Area	Study Region	Study Design	Sample size	Good level of knowledge % (95%CI)	Good practice%(95%CI)	Study quality
YOHANNES GODIE	2020	20/5/2021	Addis Ababa	Addis Ababa	Cross-sectional	420	43.1	27.1	Low risk
*Asres Bedaso*	***2018***	20/5/2021	Aleta Wondo	Sidama	Cross-sectional	313	62.8	58	Low risk
Feven Tezera	2017	20/5/2021	Dale	Sidama	Cross-sectional	170	49.4	58.9	Low risk
WEGAYEHU ZENEB	2019	15/5/2021	Debre Brhan	Amhara	Cross-sectional	660	64.7	60	Low risk
Rajalakshmi Murugan	2015	15/5/2021	Debre Markose	Amhara	Cross-sectional	339	40		Low risk
Abebe Abate	2014	9/6/2021	Debre Markose	Amhara	Cross-sectional	345		44.6	Low risk
Kaleab Sisay	2017	11/5/2021	Eastern Ethiopia	NOT SPECIFIED	Cross-sectional	495		58.3	Low risk
Haileyesus Gedamu	2018	12/5/2021	Farta	Amhara	Cross-sectional	339	51.1	54.3	Low risk
Tesfaye Getaneh	1995	12/5/2021	Jimma	Oromia	Cross-sectional	628	76	80.1	Low risk
Dejen Getaneh	2018	08/5/2021	Debre Tabor	Amhara	Cross-sectional	105	61.14	59.44	Low risk

### Publication bias

**Fig. 2:**
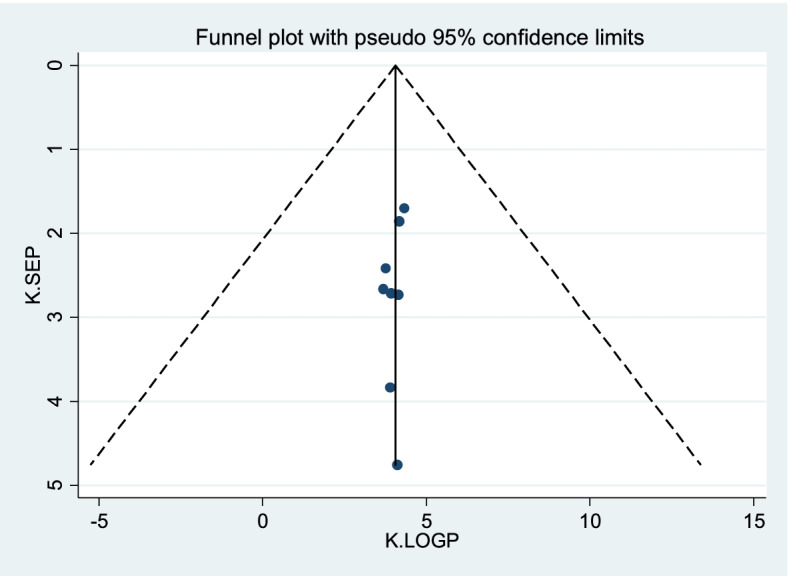
Funnel plots for publication bias of good level of knowledge towards sunshine exposure of their children among mothers in Ethiopia

**Fig. 3:**
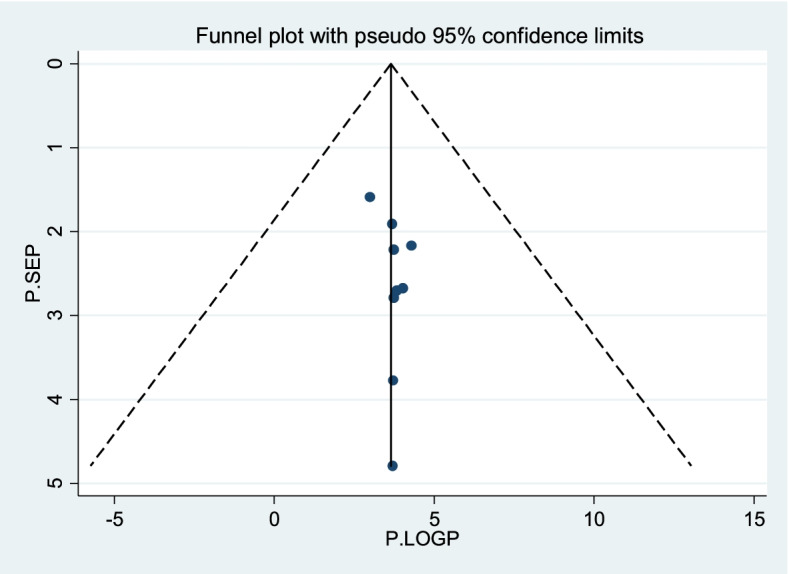
Funnel plots for publication bias of level of good practice towards sunshine exposure of their children among mothers in Ethiopia

Publication bias was assessed using a funnel plot and the Egger and Begg regression test at *P* < 0.05. There was statistical evidence of publication bias for a good level of knowledge. A funnel plot showed some asymmetrical distribution, the Begg and Egger tests were statistically significant with *P*-values = 0.621 and = 0.201 respectively (Fig. 2). There was no statistical evidence of publication bias for good practice. The Begg and Egger tests were not statistically significant with *P*-values = 0.405 and = 0.21 respectively (Fig. 3).

### Level of knowledge and practice towards sunshine exposure

**Fig. 4:**
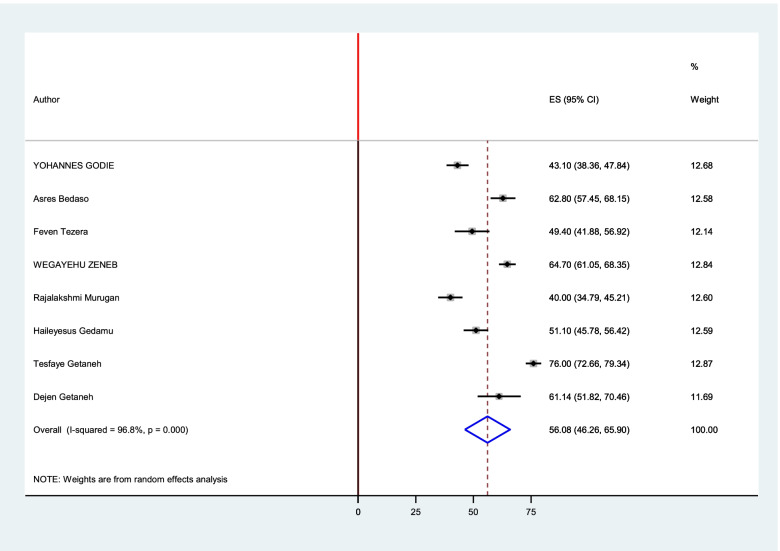
Forest plot for the pooled level of good knowledge towards sunshine exposure of their children among mothers in Ethiopia

**Fig. 5:**
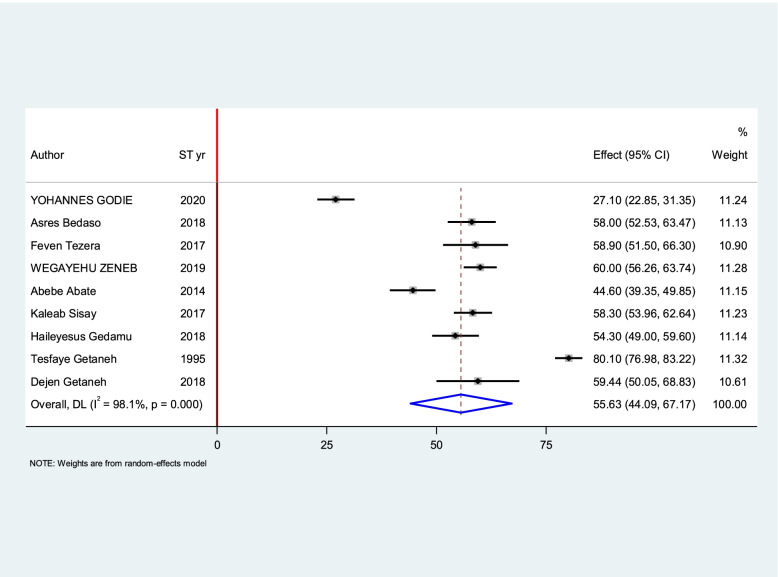
Forest plot for the pooled level of good practice towards sunshine exposure of their children among mothers in Ethiopia

The estimated overall level of good knowledge and good practice in Ethiopia is presented in a forest plot (Figs. 4 and 5). Using the random-effect model, an overall good level of knowledge was found in 56.08% (95% CI: 46.26 - 65.89%; I2 = 96.8%). The pooled estimated level of good practice towards sunshine exposure of their children among mothers in Ethiopia was found in 55.632% (95%CI: 44.091 - 67.174%; I2 = 98.2%).

### A leave-out-one sensitivity analysis

A leave-out-one sensitivity analysis was done to evaluate the effect of each study on the pooled level of good knowledge attitude and good practice by excluding each study step-by-step. The results showed that the excluded study did not bring any significant change to the estimated level of good knowledge and good practice respectively (Table 2).

**Table 2:** A-leave-out -one sensitivity analysis for mothers’ knowledge and practice towards sunshine exposure of their children in Ethiopia

Study omitted	Pooled estimate (%)	95%CI
*Knowledge related articles*		
Yohannes Godie	57.97	48.02–67.93
Asres Bedaso	55.1	43.89–66.32
Feven Tezera	56.99	46.32–67.67
Wegayehu Zeneb	54.80	42.99–66.61
Rajalakshmi Murugan	58.41	48.81–68.01
Haileyesus Gedamu	56.79	45.82–67.76
Tesfaye Getaneh	53.11	44.94–61.28
Dejen Getaneh	55.4	44.69–66.11
*Practice related articles*		
Yohannes Godie	61.43	52.84–70.01
Asres Bedaso	56.88	42.66–71.1
Feven Tezera	56.75	42.92–70.59
Wegayehu Zeneb	56.58	41.44–71.73
Kaleab Sisay	56.83	42.13–71.54
Haileyesus Gedamu	57.41	43.21–71.61
Tesfaye Getaneh	53.60	43.28–63.92
Dejen Getaneh	56.69	43.05–70.33
Abebe Abate	57.02	44.4969.54

### Subgroup analysis

The subgroup analysis based on the study region showed that the level of good knowledge was found in 54.12%(95%CI: 41.9–66.37%; I^2^ = 95.2%) in Amhara region and 56.37% (95% CI: 43.25–69.49%; I^2^ = 87.7%) in Sidama region. The pooled level of good practice in Amhara and Sidama regions found to be 54.41% (95%CI: 46.866%-53.61.941%; I^2^ = 34.1%) and 58.32%(95% CI: 53.92–62.71%; I2 = 0.0%) respectively.

### Factors associated with knowledge and practice of mothers towards sunshine exposure of their children in Ethiopia

We found insufficient data on factors affecting of knowledge and practice of mothers towards sunshine exposure to conduct meta-analyses and those who assessed the determinant factors (three primary studies) had nonhomogeneous classification (grouping) of the exposures with respect to the outcome variable (KP of sunlight exposure).

A study in Debre Markos, Amhara region revealed that mothers’ knowledge of sunlight exposure correlates with their age, educational status, family size and partner’s occupation. This study revealed that mothers in the age groups 27–32 and 33+ years were nearly five and four times more knowledgeable about sunlight exposure compared to mothers aged 15–20 years respectively. Similarly mothers whose family sized 4–6 were more knowledgeable compared to those family sized 1–3. Furthermore, the study showed that mothers who have degree and above were almost three times more knowledgeable about sunlight exposure of their child compared to those who were classified as able to read and write only [21].

Another conducted in Debre Tabor town, Amhara region indicated that mothers age less than 35 years were 1.4 times less knowledgeable about sunlight exposure compared to those mother aged 35–49 years. The study also found that singled marital status mothers were nearly three times less knowledgeable about sunlight exposure compared to those married mothers [24].

A study in Addis Abeba showed more than half (58.3%) of mothers had fear to expose their neonates to sunlight especially fear of sickness. This study also revealed neonatal age, family composition, home type, prenatal follow up, mother’s knowledge on sunlight exposure of their baby and mothers afraid of exposing their children to sunlight were the possible associated factors of sunlight exposure practice [28].

Moreover, educational status(both maternal and paternal), marital status and occupation were the important predictors of sunlight exposure practice as revealed by a study conducted in Debre Tabor town, Amhara region [24].

## Discussion

Ethiopia is a country with 13 months of sunshine and using this opportunity exposing children for morning sunshine especially during early childhood period is crucial to reduce morbidity and mortality due to vitamin-D deficiency and rickets related clinical problems [30].

Because of the limited available literature, we conducted this systematic review and meta-analysis to understand better about the mothers’ level of KP towards sunshine exposure of their children in Ethiopia. We included all available studies conducted in Ethiopia using a variety of different search engines and were also able to undertake a sub-group analysis assessing the pattern of mothers’ knowledge and practice towards sunshine exposure by geographical distribution in two regions in which more than one study available.

This study showed the highest level of good knowledge on sunshine exposure noted from Amhara region and surprisingly in the Ethiopian capital, Addis Ababa more than half of mothers hadn’t good knowledge about sunshine exposure. Though the reason why more than half of mothers hadn’t good knowledge in Addis Ababa (a city with urbanization and a large educated population) was mysterious for us, this might be due to difference in lifestyle, social composition and secio-demographic background [31].

The study showed a generally good knowledge amongst mothers varying from about 46–65% with a pooled average of nearly 56%. This finding wasn’t consistent with a previous primary study conducted in New Zealand and United Kindom (UK) where the study participants in New Zealand and UK had good knowledge compared to our finding [32, 33]. The difference in finding among the studies is an indicative of knowledge gap on sunlight exposure here in Ethiopia compared to the developed world. The finding of our study revealed that significant number of mothers hadn’t good knowledge about sunshine exposure of their children. This might be associated with quality of antenatal car (ANC) service. Studies showed quality of antenatal care service in Ethiopia was low which needs further medication on the giving clue about essential new newborn care including importance of sunshine exposure [31, 34–36]. Moreover, multi-prong strategies are required to appraise maternal knowledge about early childhood sunlight exposure.

The pooled level of good practice for sunshine exposure of their children among mothers showed that only nearly half (55%) of mothers had good practice. This might associated with negligence of providing health education about benefits, when and how to expose for sunshine. The other might be, because we do not pay attention to the things that are not payable but are very important. Furthermore, in the most Ethiopian mothers there is a traditional belief that putting the children naked in outdoor are risk for evil eye (Amharic; Buda) and which may have contributed for this finding. The subgroup analysis result showed that there was a notable consistent between regions regarding level of knowledge and practice sunlight exposure of their children.

In this study we found that, maternal age was the determinant factor of mothers’ knowledge about sunlight exposure. Two articles [21, 24] reported that, older mothers have better knowledge of sunlight light exposure of their children compared to the younger one. A good level of knowledge about childhood sunlight exposure among advanced age mothers might be attributed to their own prior experiences of childbirth. These experiences might help mothers to be aware of important information, whereby sunlight is a cost free and relatively risk-free option but is very important to prevent vitamin D deficiency related complications [37]. Therefore, health education during antenatal, natal and postnatal period for younger mothers who lacks experience of child birth could enhance knowledge about sunlight exposure.

Maternal educational level was the other determinant factor of mother’s knowledge about sunlight exposure of their children. Mothers who were educated were knowledgeable about sunning their child compared to those those uneducated [21]. This finding supported by previous studies conducted on related clinical problems in Ethiopia [38, 39]. This might be attributed to health information exposure among educated women that could be obtained from school. Furthermore, educated mothers might have less difficulty of understanding the information received from healthcare workers as compared to their uneducated counterparts [40]. Furthermore, educated mothers can seek, read and understand health messages from communication channels, such as government and other social Medias.

This study also found that educational status (both maternal and paternal), age, marital status and occupation have a significant association with maternal sunning practice. This finding wasn’t supported by a primary study conducted in Turkey in which there was no significant correlation between mothers’ sunning behavior and age, education level [41]. This difference might be clarified due to differences in type, educational status, and level of awareness about sunlight exposure among study populations. Indeed, methodological difference between studies might have a considerable significance for the observed difference.

In our study, the variation between studies resulted in a significant between-study heterogeneity. To assess this further, we used a random-effect model as well as a leave one-out-one sensitivity analysis. The results showed that the estimated pooled level of good knowledge and good practice was robust and not dependent on a single study. We assessed the possible source variability by sub-group analysis using study region. The high heterogeneity might be due to differences in the sample population between studies and the short study period.

Moreover, our finding will have health policy and clinical implications for maternal and child health care services in Ethiopia. The Ethiopian public health policies must address the need for childhood sunlight exposure and this national public health messages should be spread using various platforms (i.e., social media, health institutions, universities, schools) to increase awareness among all sub-groups of the population since protecting children from preventable clinical problems by practicing such essential care should be a matter of everyone. We also call for national healthcare authorities of Ethiopia and other host counties to give an emphasis for policies and programs targeting community health education and appropriate information dissemination about child care including sunlight exposure in various circumstances, such as during perinatal care, immunization, integrated management of newborn and childhood illness service.

### Strength and limitation of the study

The strengths of the study included the comprehensive search strategy through the different datasets to estimate the national level of mothers’ knowledge and practice towards sunshine exposure of their children, the involvement of more than one assessor in the quality evaluation and the use of the appraisal process using JBI-MAStARI. Our study also had some limitations. These included the fact that measurements for the level of knowledge and practice were taken from each primary study and operational definitions may have differed between the studies. The absence of a previous similar study makes it difficult to compare our findings with other findings. In addition, this meta-analysis didn’t pool the determinant factors that affect KP of mother’s sunshine exposure of their children. Finally, our search strategy found limited studies and this calls into question the national representativeness of our study.

## Conclusion

Our systematic review and meta-analysis showed that there was a quite gap in maternal knowledge and practice towards sunshine exposure of their children in Ethiopia. This is important information and requires that stakeholders should give attention about working on it using various platforms (i.e., social media, health institutions, universities, schools) to increase awareness among all sub-groups of the population. Further research is required to determine the mothers’ level of KP on sunshine exposure in other regions of the country and more information about why this gap exists.

## Data Availability

All data about this study are contained and presented in this document.

## Abbreviations

ANC: Antenatal Care
CI: Confidence Interval
DARE: Database of Abstracts of Review of Effects
EMBASE: Excerpta Medica database
KP: Knowledge and Practice
JBI: The Joanna Briggs Institute
PRISMA: Preferred Reporting Items for Systematic Review and Meta-Analysis
SNNP: South Nations Nationalities and Peoples Region
UK: United Kingdom
